# Placenta previa with posterior extrauterine adhesion: clinical features and management practice

**DOI:** 10.1186/s12893-020-01027-9

**Published:** 2021-01-06

**Authors:** Yoshikazu Nagase, Shinya Matsuzaki, Masayuki Endo, Takeya Hara, Aiko Okada, Kazuya Mimura, Kosuke Hiramatsu, Aiko Kakigano, Erika Nakatsuka, Tatsuya Miyake, Tsuyoshi Takiuchi, Yutaka Ueda, Takuji Tomimatsu, Tadashi Kimura

**Affiliations:** 1 Department of Obstetrics and Gynecology, Osaka University Graduate School of Medicine, 2-2 Yamadaoka, Suita, Osaka 565-0871 Japan; 2 Division of Gynecologic Oncology, Department of Obstetrics and Gynecology, University of Southern California, Los Angeles, CA USA; 3 Department of Health Science, Osaka University Graduate School of Medicine, Osaka, Japan; 4 Department of Obstetrics and Gynecology, Aizenbashi Hospital, Osaka, Japan; 5 Department of Obstetrics and Gynecology, National Cerebral and Cardiovascular Center, Osaka, Japan

**Keywords:** Adhesion, Endometriosis, Magnetic resonance imaging, Bakri balloon, Placenta previa

## Abstract

**Background:**

A diagnostic sign on magnetic resonance imaging, suggestive of posterior extrauterine adhesion (PEUA), was identified in patients with placenta previa. However, the clinical features or surgical outcomes of patients with placenta previa and PEUA are unclear. Our study aimed to investigate the clinical characteristics of placenta previa with PEUA and determine whether an altered management strategy improved surgical outcomes.

**Methods:**

This single institution retrospective study examined patients with placenta previa who underwent cesarean delivery between 2014 and 2019. In June 2017, we recognized that PEUA was associated with increased intraoperative bleeding; thus, we altered the management of patients with placenta previa and PEUA. To assess the relationship between changes in practice and surgical outcomes, a quasi-experimental method was used to examine the difference-in-difference before (pre group) and after (post group) the changes. Surgical management was modified as follows: (i) minimization of uterine exteriorization and adhesion detachment during cesarean delivery and (ii) use of Nelaton catheters for guiding cervical passage during Bakri balloon insertion. To account for patient characteristics, propensity score matching and multivariate regression analyses were performed.

**Results:**

The study cohort (*n* = 141) comprised of 24 patients with placenta previa and PEUA (PEUA group) and 117 non-PEUA patients (control group). The PEUA patients were further categorized into the pre (*n* = 12) and post groups (*n* = 12) based on the changes in surgical management. Total placenta previa and posterior placentas were more likely in the PEUA group than in the control group (66.7% versus 42.7% [*P* = 0.04] and 95.8% versus 63.2% [*P* < 0.01], respectively). After propensity score matching (*n* = 72), intraoperative blood loss was significantly higher in the PEUA group (*n* = 24) than in the control group (*n* = 48) (1515 mL versus 870 mL, *P* < 0.01). Multivariate regression analysis revealed that PEUA was a significant risk factor for intraoperative bleeding before changes were implemented in practice (*t* = 2.46, *P* = 0.02). Intraoperative blood loss in the post group was successfully reduced, as opposed to in the pre group (1180 mL versus 1827 mL, *P* = 0.04).

**Conclusions:**

PEUA was associated with total placenta previa, posterior placenta, and increased intraoperative bleeding in patients with placenta previa. Our altered management could reduce the intraoperative blood loss.

## Background

Placenta previa (PP) is a risk factor for preterm birth and postpartum hemorrhage (PPH); approximately half of PP cases result in PPH [1]. The risk of massive hemorrhage in patients with PP varies according to co-existing risk factors. For instance, PP is the most significant risk factor for placenta accreta spectrum (PAS) disorders, and if PP is complicated by PAS, the surgical morbidity and mortality, mean blood loss (1200–3000 mL), and hysterectomy rates (3–42%) increase dramatically [2–4]. Several reports have suggested that preoperative assessment of PAS disorders and a multidisciplinary surgical approach are essential to improve outcomes and lower complication rates for cesarean delivery (CD) in PAS patients [5–7]. Therefore, knowing the risk factors of intraoperative bleeding and carefully assessing the surgical management of patients with PP are clinically essential.

We previously reported a horizontal cervix sign on magnetic resonance imaging (MRI) in patients with PP that suggests posterior extrauterine adhesion (PEUA; Additional file 1: Figure S1) [8]. In our previous study, approximately 20% of patients with PP in the cohort had PEUA [8]. Non-pregnant women with PEUA or cul-de-sac obliteration associated with endometriosis experienced increased surgical morbidity, such as more frequent ureteral injuries and prolonged operative times [9, 10]. Therefore, it is hypothesized that PEUA may be associated with increased intraoperative blood loss during CD; however, the surgical outcomes of CD in patients with PP and PEUA have not been examined. Moreover, while the risk factors of PAS have been widely determined, whether PEUA is associated with PAS has not been investigated [4]. Since PP is a major risk factor of PPH, it is important to examine whether PEUA increases the risk of massive hemorrhage in patients with PP.

From June 2017, we recognized that PEUA is a possible risk factor of massive hemorrhage during CD in patients with PP. Thus, we revised our management strategy, aiming to improve surgical outcomes. The primary aim of the present study was to investigate the clinical characteristics of PP with PEUA. The secondary aim was to determine whether the changes in practice improved the surgical outcomes after CD.

## Methods

### Data source and eligibility

This single institutional, retrospective, observational study was performed at a tertiary referral medical center. Patients with PP (including a low-lying placenta) who underwent CD between January 2014 and May 2019 at the Osaka University, and whose operation records were available, were examined. A portion of the same dataset was used in our previous retrospective study that focused on identifying MRI features that predicted PEUA or the side effects of uterine compression sutures [8, 11]. This study was approved by the Osaka University Research Ethics Committee (Approval No. 19467, February 13, 2020) and was conducted in accordance with the Declaration of Helsinki. Informed consent was not required from the patients because of the retrospective nature of this study, which was based on computerized data and anonymous selection criteria.

### Clinical information

We reviewed the intraoperative findings in the surgical records, and based on the presence of PEUA, categorized the patients into the PEUA and control groups. As in our previous study [8], patients were included in the PEUA group if their records described an adhesion between the posterior extrauterine wall and the small bowel, colon, rectum, ovary, or pelvic wall. Representative images of PEUA are shown in Additional file 2: Figure S2a and S2b. Women who delivered before 22 weeks of gestation and those with huge myomas were excluded. The following clinical characteristics and outcomes were analyzed: (1) maternal age at delivery, (2) body mass index, (3) parity, (4) in vitro fertilization-embryo transfer, (5) gestational age at delivery, (6) number of previous CDs, (7) type of PP, (8) location of the placenta, (9) cervical length measured at 27–32 weeks of gestation, (10) perinatal complications (such as preeclampsia, gestational diabetes mellitus, and fetal growth restriction), (11) intraoperative blood loss during CD, (12) additional treatments for hemostasis (such as intrauterine balloon tamponade [IUBT], uterine compression sutures [UCS], interventional radiology [IVR], and hysterectomy), and (13) blood transfusion.

For better estimation of the intraoperative blood loss, a vinyl sheet was placed under the patient (Additional file 3: Figure S3); this vinyl sheet had two pockets for the patient’s sides and a pocket with a tube and bottle for collecting the vaginal bleeding. The amount of blood loss was then calculated after excluding the estimated amount of the amniotic fluid.

### Study definition

Ultrasound findings indicative of PAS, such as lacunae, loss of retroplacental clear space, irregularity of uterine-bladder interface, and the smallest sagittal myometrial thickness, were used for the screening of PAS [12–15]. Since the cost of an MRI is relatively reasonable (approximately US$300) in Japan [14], patients were evaluated by MRI when they met the following criteria: (1) presenting with PP and having risk factors for PAS (such as having an advanced maternal age [> 40 years], in vitro fertilization, prior CD, or anterior placenta), (2) presence of ultrasound indicators for PAS, and (3) difficultly in evaluating the risk of PAS by ultrasonography due to a posterior placenta.

After the MRI images were obtained, the patients were categorized into eight groups according to the presence or absence of PAS findings, and an antenatal diagnosis of PAS was made as described previously (Additional file 4: Table S1) [14]. Groups 1–5 were antenatally diagnosed as patients with PAS. In this study, we secondarily evaluated the diagnostic accuracy of the horizontal cervix sign for the presence of PEUA using MRI images taken for the preoperative diagnosis of PAS.

**Fig. 1:**
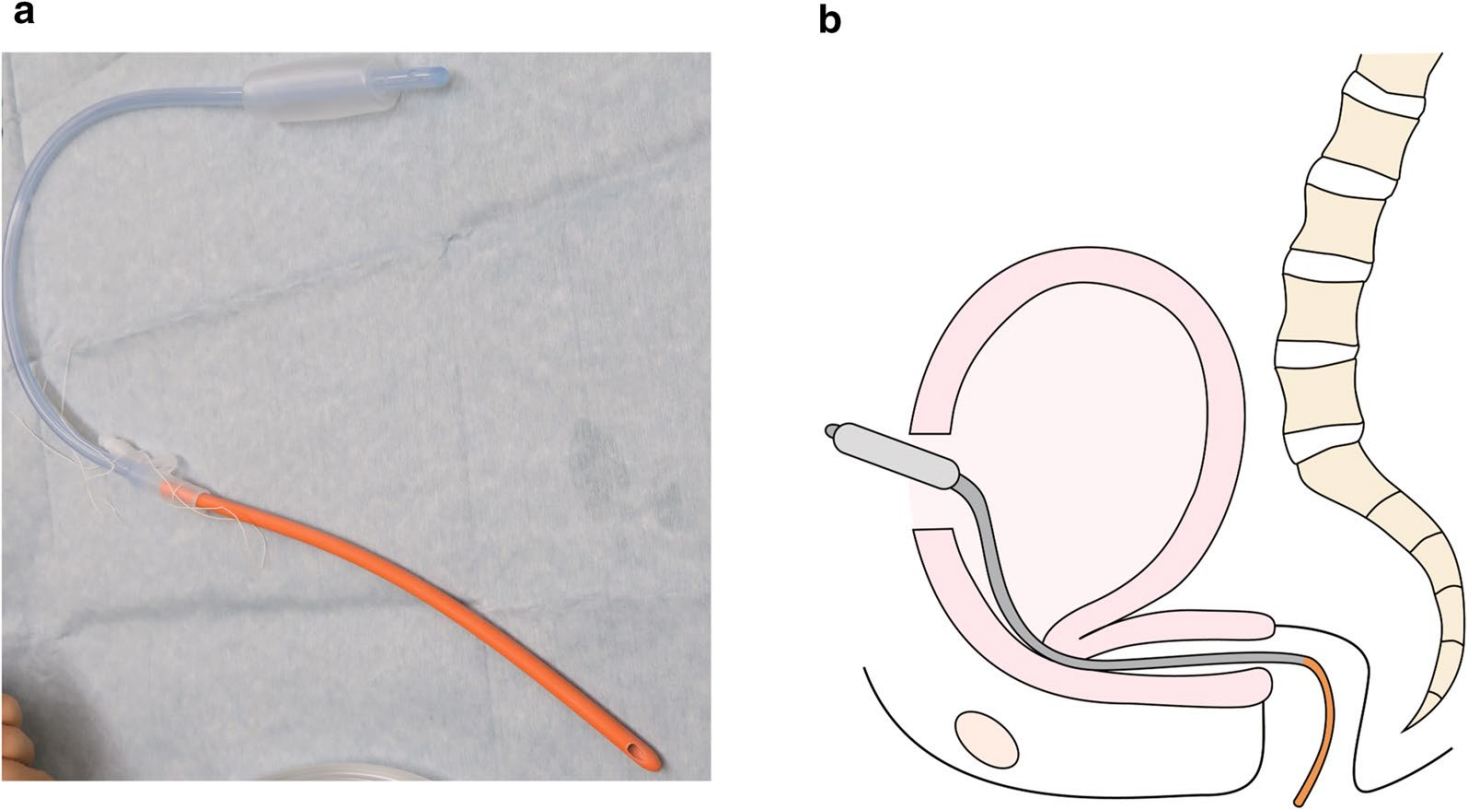
Intrauterine balloon insertion method using a Nelaton catheter as a guide. **a** A 26-French Nelaton catheter is used and connected to the blood drainage port of a Bakri balloon. The Nelaton catheter is inserted from the uterine incision to the vagina through the cervix. **b** Insertion of a Bakri balloon using a Nelaton catheter as a guide for cervical passage. Safe and easy placement of an intrauterine balloon, even in a strongly bent uterus, is possible using a Nelaton catheter with appropriate stiffness as a guide

Beginning in June 2017, we began to use this horizontal cervix sign for evaluating PEUA when MRI was performed to determine the presence of PAS. We also implemented the following changes in the management of patients suspected of PP with PEUA: (1) careful intraoperative assessment of PEUA, (2) minimization of uterine exteriorization and adhesion detachment, and (3) if PPH was observed, insertion of a Bakri balloon transabdominally using a Nelaton catheter as a guide for cervical passage, as previously described (Fig. 1) [16–18]. IUBT and UCS were performed as previously described [11, 19, 20].

### Statistical consideration

A quasi-experimental method was used to assess the relationship between changes in practice and the surgical outcomes, by examining the difference-in-difference before (the pre group) and after (the post group) the changes in practice were implemented [21].

To assess whether PEUA was associated with intraoperative bleeding in patients with PP, propensity score matching and multivariate regression analysis were performed [22, 23]. The Statistical Package for Social Sciences (IBM SPSS, version 26.0, Armonk, NY) was used for propensity score matching analysis. Propensity score matching was used to reduce the imbalance between the PEUA and control groups. The propensity scores were estimated using the following demographic variables: age, body mass index, previous CD (yes versus no), placenta location (anterior versus posterior), type of PP (total versus partial/marginal/low-lying), and placenta accreta (yes versus no). The control group was generated automatically to avoid selection biases, and the PEUA group was matched with the control group at a ratio of 1:2 [22, 23].

Multivariate linear regression analysis was performed to identify the factors associated with increased intraoperative bleeding. Predictor variables included PEUA, PAS, advanced maternal age, in vitro fertilization-embryo transfer, prior CDs, and the type of CD (elective or emergency).

All statistical analyses were performed using JMP Pro version 14.0.0 (SAS Institute, Cary, NC, USA). Continuous variables were analyzed using the Student’s *t*-test or the Mann–Whitney *U* test. Categorical variables were analyzed using the Chi-square or the Fisher’s exact test. For comparisons among the control, pre, and post groups, *P*-values were calculated and corrected using a Dunn–Bonferroni test. *P*-values < 0.05 indicated statistical significance.

## Results

### Retrospective analysis of PP with and without PEUA

A total of 141 patients with PP were identified during the study period; of these, 24 presented with PEUA (the PEUA group), while 117 presented without PEUA (the control group). The patients’ demographics are shown in Table 1. Total PP (66.7% versus 42.7%, *P* = 0.04) and posterior placentas (95.8% versus 63.2%, *P* < 0.01) were more likely in the PEUA group than in the control group. Although the cervical length measured at 27–32 weeks of gestation was significantly longer in the PEUA group than in the control group (56.1 mm versus 36.1 mm, *P* < 0.01), the preterm birth rate was similar between the two groups (50.0% versus 57.3%, *P* = 0.65). Intraoperative blood loss (1,515 mL versus 1,500 mL, *P* = 0.65) and transfusion rate (8.3% versus 12.0%, *P* = 0.99) was similar between the two groups. The hysterectomy rate (4.2% versus 23.9%, *P* = 0.03) was significantly higher in the control group than in the PEUA group due to the imbalance of PAS rate.

**Table 1:** Baseline characteristics and surgical outcomes of patients with placenta previa

	PEUA	Control	*P* value
Number of cases	24	117	
PAS	1 (4.2)	28 (23.9)	**0.03**
Maternal age, years (range)	34 (27–48)	35 (26–53)	0.81
BMI at delivery (range)	21.8 (19.1–27.2)	23.5 (17.6–41.0)	** < 0.01**
Parity, primipara	20 (83.3)	49 (41.9)	** < 0.01**
IVF-ET status	9 (37.5)	22 (18.8)	**0.04**
GA at delivery, wks (range)	36.5 (29–37)	36 (29–39)	0.96
PTB	12 (50.0)	67 (57.3)	0.65
Prior CDs, none	21 (87.5)	97 (82.9)	0.77
Type of PP			
Total PP	16 (66.7)	50 (42.7)	**0.04**
Others^a^	8 (33.3)	67 (57.3)	
Location of placenta			** < 0.01**
Anterior or central	1 (4.2)	43 (36.8)	
Posterior	23 (95.8)	74 (63.2)	
Cervical length^b^, mm (range)	56.1 (38.8–68.0)	36.1 (18.9–56.5)	** < 0.01**
Perinatal complications			
Preeclampsia	1 (4.2)	1 (0.9)	0.31
GDM	3 (12.5)	8 (6.8)	0.40
FGR	0 (0)	6 (5.1)	0.59
Surgical outcomes			
Intop blood loss (range)	1515 (500–4500)	1500 (200–4300)	0.65
Transfusion	2 (8.3)	14 (12.0)	0.99
Hysterectomy	1 (4.2)	28 (23.9)	**0.03**
For PAS	1	26	** < 0.01**

Number (% per group) or median is shown. Significant *P*-values are emboldened

*PP* placenta previa, *PEUA* posterior extrauterine adhesion, *Intop* intraoperative, *GA* gestational age, *PTB* preterm birth, *PAS* placenta accreta spectrum, *BMI* body mass index, *IVF-ET* in vitro fertilization-embryo transfer, *wk* week, *CD* cesarean delivery, *GDM* gestational diabetes mellitus, *FGR* fetal growth restriction

^a^Others included partial PP, marginal PP, and low-lying placenta

^b^Cervical length was measured at 27–32 weeks of gestation with transvaginal ultrasound scan

Among the 29 cases with PAS, 26 were diagnosed antenatally. Furthermore, 27 of these 29 patients underwent hysterectomy following CD, while the remaining 2 [24, 25] with placenta percreta underwent conservative management.

### Surgical outcomes between the PEUA and the control groups

In the propensity score matching model (Table 2), all the measured covariates (except for the rate of PAS) were matched without significant clinical imbalance (all except for PAS, *P* > 0.05; PAS: *P* = 0.03) between the PEUA and control groups. In the matched cohort (*n* = 72), the amount of intraoperative blood loss was significantly higher in the PEUA group (*n* = 24), than in the control group (*n* = 48) (1515 mL [range 500–4500 mL] versus 870 mL [range 200–2900 mL], *P* < 0.01).

**Table 2:** Patient demographics and surgical outcomes before and after propensity score matching

	After PS matching		
	PEUA	Control	*P* value
Number of cases	24	48	
PAS	1 (4.2)	13 (27.1%)	**0.03**
Maternal age, years (range)	34 (27–48)	35.5 (26–42)	0.565
BMI at delivery (range)	21.8 (19.1–27.2)	23.4 (18.8–32.0)	0.08
IVF-ET status	9 (37.5)	12 (25.0%)	0.286
GA at delivery, wks (range)	36.5 (29–37)	36 (30–38)	0.571
Prior CDs, None	21 (87.5)	38 (79.2%)	0.999
Type of PP			0.447
Total PP	16 (66.7)	22 (45.8%)	
Others^a^	8 (33.3)	26 (54.2%)	
Location of placenta			0.14
Anterior or central	1 (4.2)	15 (31.3%)	
Posterior	23 (95.8)	33 (58.7%)	
Surgical outcomes			
Intop blood loss (range)	1515 (500–4500)	870 (200–2900)	** < 0.01**
Transfusion	2 (8.3)	2 (4.2%)	0.60
Hysterectomy	1 (4.2)	12 (25.0%)	0.05
For PAS	1	11	0.05

Number (% per group) or median is shown. Significant *P*-values are emboldened

*PEUA* posterior extrauterine adhesion, *PP* placenta previa, *Intop* intraoperative, *PS* propensity score, *GA* gestational age, *PTB* preterm birth, *PAS* placenta accreta spectrum, *BMI* body mass index, *IVF-ET* in vitro fertilization-embryo transfer, *wk* week, *CD* cesarean delivery

^a^Others included partial PP, marginal PP, and low-lying placenta

The results of propensity score matching suggested that PEUA was associated with increased intraoperative bleeding. Multivariate linear regression analysis, which was performed to enhance the robustness of our results, revealed that PEUA was associated with increased intraoperative bleeding before the changes in practice were implemented (*t* = 2.46, *P* = 0.02, Table 3).

**Table 3:** Multivariate linear regression analysis of the predictors for blood loss during delivery

Variable	Non-SCE		SCE	*t*-value***	*P* value	95% CI	
	B*	SE	Beta**			Inferior	Superior
**PEUA**^**a**^	**266.2**	**108.3**	**0.2**	**2.46**	**0.02**	**52.0**	**480.4**
PAS	29.7	78.2	0.033	0.38	0.7	− 124.9	184.4
AMA^b^	111.7	62.4	0.15	1.79	0.08	− 11.8	235.2
IVF-ET	− 9.96	74.6	− 0.011	− 0.13	0.89	− 157.6	137.7
Prior CDs	− 125.0	85.2	− 0.13	− 1.47	0.15	− 293.6	43.6
Type of CD	91.4	63.5	0.12	1.44	0.15	− 34.4	217.1

Bold indicates statistical significance

*SCE* standardized coefficients, *SE* standard error, *CI* confidence interval, *PAS* placenta accreta spectrum, *AMA* advanced maternal age, *IVF-ET* in vitro fertilization-embryo transfer, *CD* cesarean delivery, *inferior* inferior limit, *superior* superior limit

*B quantifies the impact of each variable on the intraoperative blood loss during CD. **Beta refers to the change in standard deviation for the intraoperative blood loss for an increment of one standard deviation of the explanatory variable. ***t-value refers to the significance level of B

^a^PEUA indicates patients with posterior extrauterine adhesion in the Pre group

^b^AMA indicates over 35 years old

### Surgical outcomes pre- and post-change in the practice groups

To assess the effect of altered management on surgical outcomes, the patient characteristics and surgical outcomes were compared between the pre (*n* = 12) and post groups (*n* = 12). There were no significant differences in the patient characteristics between the two groups (Additional file 5: Table S2). Furthermore, IUBT with a Nelaton catheter was more likely to be performed in patients in the post group than in those in the pre group (66.7% [8/12] versus 16.7% [2/12], *P* = 0.04) (Table 4). This trend was also observed in the cohort without PAS (post group: 66.7% [8/12], pre group: 18.2% [2/11]; *P* = 0.04).

**Table 4:** Comparison of surgical outcomes in placenta previa with posterior extrauterine adhesion before and after changes in practice in June 2017

	Pre	Post	*P* value
Number of cases	12	12	
PAS	1 (8.3)	0 (0)	0.99
Total blood loss during delivery, mL (range)	1827 (500–4500)	1180 (950–1800)	**0.02**
Additional treatment			
IUBT	5 (41.7)	8 (66.7)	0.41
Using a Nelaton catheter^a^	2 (16.7)	8 (66.7)	**0.04**
UCS	4 (33.3)	1 (8.3)	0.32
IVR	0 (0)	0 (0)	NA
Hysterectomy	1 (8.3)	0 (0)	0.99
Blood transfusion	2 (16.7)	0 (0)	0.48

Number (% per group) or median is shown. Significant *P*-values are emboldened

*Pre* pre-change posterior extrauterine adhesion group, *Post* post-change posterior extrauterine adhesion group, *PAS* placenta accreta spectrum, *IUBT* intrauterine balloon tamponade, *UCS* uterine compression sutures, *IV*R interventional radiology, *NA* not applicable

^a^Patients treated with IUBT using the insertion method using a Nelaton catheter as a guide for cervical passage

**Fig. 2:**
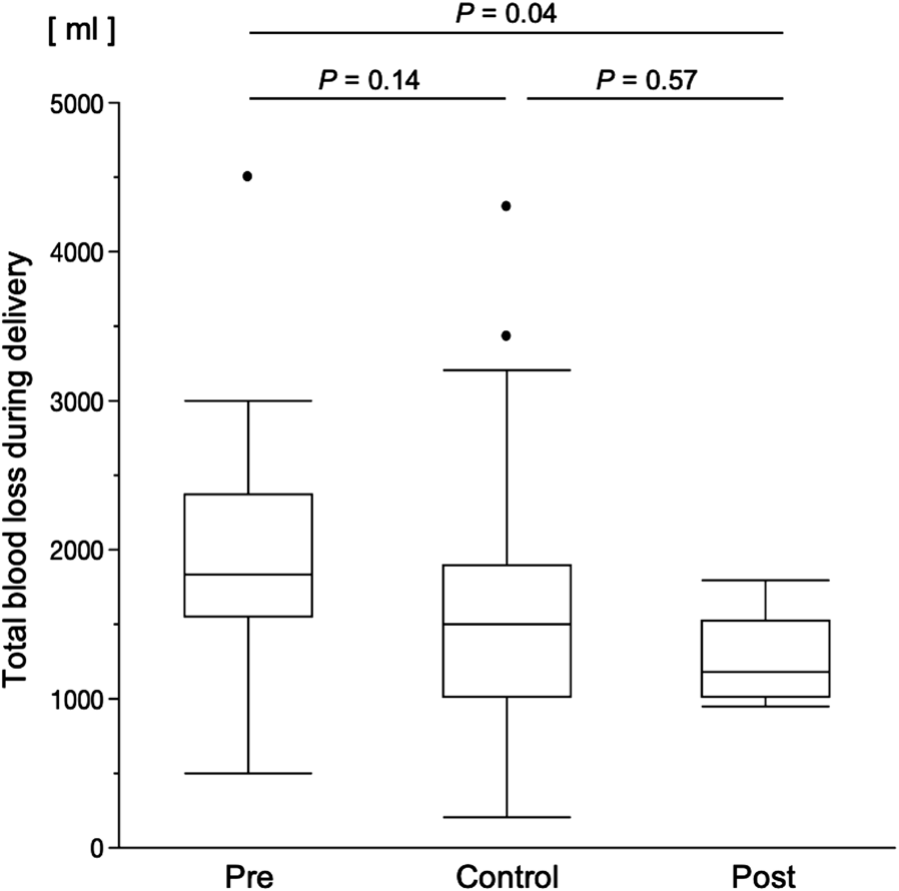
Assessment of the relationship between changes in practice and intraoperative blood loss. Abbreviations: *Pre* pre-change posterior extrauterine adhesion group; *Post* post-change posterior extrauterine adhesion group

Albeit statistically non-significant, the intraoperative blood loss during CD (Fig. 2) tended to be higher in the pre group than in the post group (1827 mL [range 500–4500 mL] versus 1500 mL [range 200–4300 mL], *P* = 0.14). However, the blood loss was lower in the post group (1180 mL, range 950–1800 mL; *P* = 0.04) than in the pre group.

### Diagnostic accuracy of the horizontal cervix sign for PEUA

Around 82 patients (PEUA group: *n* = 23, control group: *n* = 59) underwent MRI for preoperative evaluation of PAS. The horizontal cervix signs were observed in 26 patients, of which 20 and 6 were in the PEUA and control groups, respectively. The diagnostic accuracy of the horizontal cervix sign for PEUA was as follows: sensitivity: 87.0%, specificity: 89.8%, positive predictive value: 76.9%, and negative predictive value: 94.6%. In the post group (*n* = 12), PEUA was suspected antenatally in 10 (83.3%) patients with a positive horizontal cervix sign, while two patients were diagnosed with PEUA during CD. Altered surgical management was successfully adopted in all patients in the post group (*n* = 12).

The causes of PEUA were assessed in 24 patients (Additional file 6: Table S3). Among these, uterine exteriorization was performed in 17 (70.8%) patients in whom endometriosis was confirmed intraoperatively. Among the remaining 7 patients, i.e., the non-uterine exteriorization group, two patients had undergone a prior surgery for endometriosis and five had no prior surgical or medical history.

## Discussion

Our study had two key findings. First, PP with PEUA was associated with intraoperative bleeding. Second, our changes in practice were associated with decreased intraoperative blood loss in patients with PP and PEUA.

Our results revealed that patients with PP and PEUA were at a higher risk of increased intraoperative blood loss, especially before the changes in practice were implemented. This finding suggests that it is essential to understand the underlying mechanism connecting PEUA with increased intraoperative blood loss, as well as the methods to control PPH in patients with PP and PEUA.

Various hemostatic procedures, including IUBT, UCS, IVR, and hysterectomy, could be performed in patients with PP. Of these, UCS can be performed without specific equipment, but it requires exteriorization of the uterus. Prior to the change in our management practices, we performed hemostatic procedures in patients with PP and PEUA that required exteriorization of the uterus, such as UCS and vertical compression sutures in the lower uterine segment. We found that adhesions of the intestine and/or ovaries to the posterior extrauterine wall often made these procedures impossible or substantially longer. In short, we found that using UCS for patients with PEUA required uterine exteriorization, adhesion detachment, and more time to achieve hemostasis.

**Fig. 3:**
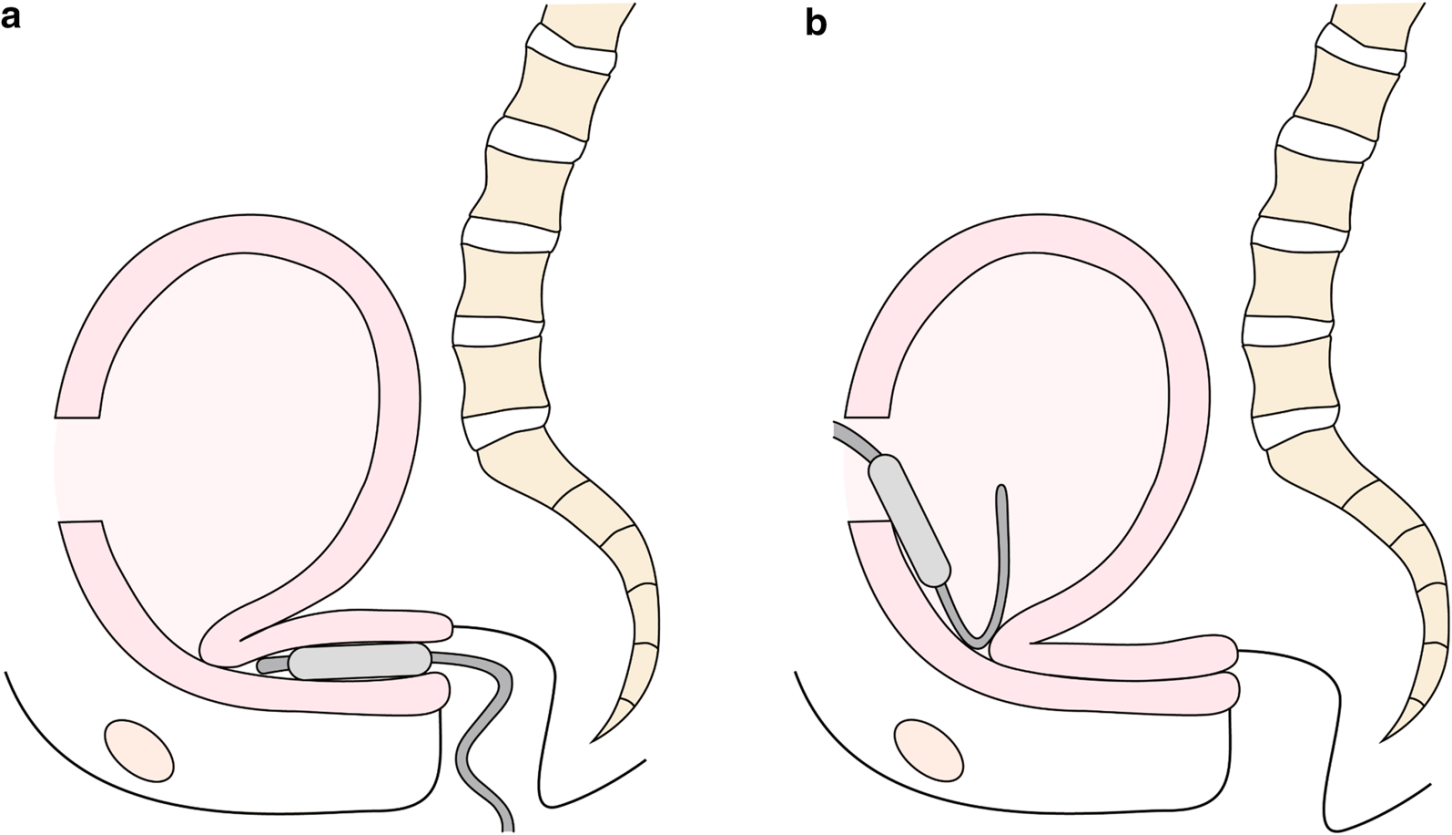
Illustration demonstrating the difficulty of Bakri balloon insertion in patients with posterior extrauterine adhesion. Intrauterine balloon tamponade without using a Nelaton catheter as a guide requires additional time for **a** transvaginal and **b** transabdominal placement because the balloon catheter is bent by strong retroflexion of the uterus and the longer cervical length

IUBT without a Nelaton catheter also required additional time for placement in patients with PEUA because the balloon catheter was bent by strong retroflexion of the uterus and a longer cervical length (absolute cervical length: 20 mm; Table 1). Exteriorization of the uterus is necessary to correct the strong retroflexion; however, it results in a higher risk of additional bleeding from the posterior extrauterine wall due to unavoidable adhesion detachment. For this reason, it was difficult to place the intrauterine balloon transvaginally or transabdominally without uterine exteriorization (Fig. 3).

Following implementation of the changes in our practice, we utilized a Nelaton catheter as a guide for cervical passage during the insertion of a Bakri intrauterine balloon. This method has been reported by Matsubara et al. as a prompt and easy method for the insertion of an intrauterine balloon during CD in patients with PP and massive bleeding [16–18, 26]. In the present study, this insertion method facilitated the placement of the intrauterine balloon without exteriorization of the uterus and allowed passage through the narrow long cervix (Fig. 1). Because of these changes in practice, the intraoperative bleeding in patients with PP and PEUA was significantly reduced.

Endometriosis may be one of the risk factors of PEUA. In this study, we speculated that endometriosis was involved in ~ 75% of the patients with PP and PEUA. Several systematic reviews and retrospective studies have identified endometriosis, including endometrioma, as a significant risk factor for PP [27–29]. Because the development of assisted reproductive technologies has increased pregnancy rates in women with endometriosis, the number of patients with PP and endometriosis may increase in the future [30, 31]. Therefore, it is feared that the number of patients with PP and PEUA, which are high-risk patients, will increase. For these reasons, we believe our findings will benefit clinicians who treat high-risk patients with PP.

To the best of our knowledge, there have been no reports investigating the relationship between endometriosis and PP with PEUA. Previous studies examining the association between endometriosis and PP have proposed that endometriotic lesions in the uterus may reduce uterine contractility [32], and uterine dysperistalsis may cause abnormal blastocyst implantation, resulting in PP [32–34]. Here, we reported more cases of total PP and posterior placenta in PP patients with PEUA than in those without PEUA, which may also be related to dysperistalsis of the uterus due to endometriosis.

Recent studies have shown the usefulness of ultrasonographic findings in early pregnancy to diagnose PAS or stratify the surgical outcomes of women with PAS. In a retrospective study investigating 188 women with PP with prior CD has shown that ultrasound performed between 11 and 14 weeks’ gestation had a good diagnostic accuracy for detecting PAS [35]. In another retrospective study that assessed 187 women with PP with prior uterine surgery by transvaginal ultrasonography, the presence of cross-over sign (COS; an ectopic gestational sac and endometrial line [36]) between 5 and 7 weeks’ gestation was associated with adverse surgical outcomes such as massive bleeding, incidence of surgical complications, and admission to the intensive care unit [37]. Since MRI or ultrasonographic findings of PEUA in early pregnancy were not examined in this study, future studies to examine MRI or ultrasonographic findings of PEUA in early pregnancy are warranted.

Preoperative detection of PEUA using the horizontal cervix sign on MRI may have played a role in reducing intraoperative bleeding after changes in our practice were implemented, because 10 out of 12 patients were suspected of PEUA antenatally. Since MRI examinations cannot be performed for PEUA screening due to the high cost, it is expected that transvaginal ultrasonography will be used to identify horizontal cervixes in the future.

We observed a difference in the diagnostic accuracy of the horizontal cervix sign for posterior extrauterine adhesion between this study (sensitivity: 87.0%, specificity: 89.8%, positive predictive value: 76.9%, and negative predictive value: 94.6%) and our previous study (sensitivity: 81.0%, specificity: 89.3%, positive predictive value: 68.0%, and negative predictive value: 94.3%) [8]. We have two hypotheses regarding this matter. In our previous study [8], we have identified that a positive predictive value of the horizontal cervix sign was higher if an MRI examination was conducted at ≤ 32 weeks of gestation. Approximately 30% of the patients were evaluated by MRI after 33 weeks of gestation in our previous study [8]; however, in the current study, only 10% of the patients were evaluated by MRI after 33 weeks of gestation.

Another possible reason may be due to the difference in the number of institutions where the study was performed. Our previous study was conducted at two tertiary centers, while the current study was conducted at one center. To avoid bias due to limited cases and institutions, a multicenter study is warranted in the future.

The strength of our study is that this is likely the first report to focus on the clinical features and outcomes of PP with PEUA. We performed a careful evaluation and changed our management practices for PP with PEUA, and successfully reduced the intraoperative bleeding during CD.

Our study also has several limitations. First, since it was a single-center retrospective study with a relatively small sample size, it has an unmeasured bias inherent to this type of study. Additionally, a heterogeneity was observed across the patient backgrounds. We performed a propensity score matching analysis to match the patient characteristics, but the rate of PAS could not be matched due to the small sample size. Nevertheless, we believe that an analysis of this sample size has sufficient clinical value because PP with PEUA is relatively rare. To enhance the strength of our findings, a study with a larger number of patients is warranted.

Secondly, it is important to note that the population of PP patients with PEUA in our institution may be higher than that of a general hospital. Our university hospital receives referrals for many pregnant women who conceived by IVF-ET or who had a history of surgery for ovarian endometrioma. Third, though a majority of the PEUA cases in this study were associated with endometriosis, no cases presented with an endometrioma. Moreover, these diagnoses are dependent on intraoperative findings during CD. In general, an intraoperative finding of endometriosis should be confirmed by histopathological analysis. However, we did not perform histopathological analyses to avoid unnecessary bleeding during biopsies. Therefore, the diagnosis of endometriosis in this study was ambiguous, and a possibility of severe bias should be considered. To reduce the ambiguity in endometriosis assessment by surgeons, typical intraoperative findings are shown in Additional file 2: Figure S2a and S2b.

Fourth, the diagnostic accuracy of the horizontal sign for PEUA was different between our previous study and the current study; thus, a study with a large number of cases and a unified indication of MRI is warranted for examining the diagnostic accuracy. Fifth, although we assessed the presence of PAS findings and the horizontal cervix sign for PEUA using MRI in this study, we should note that MRI is difficult to perform because of its high costs in most countries. Since an ultrasonographic finding for PEUA was not identified in this study, future studies are required to identify one. Notably, we believe that identifying an ultrasonographic finding for PEUA in early pregnancy is useful and needs to be determined.

Lastly, this study included very few PAS cases, and we could not examine or propose treatment practices for PAS patients with adhesions. We also could not examine whether PEUA was a risk factor of PAS due to the small sample size. Therefore, to enhance the strength of our findings, a study with a larger number of patients should be conducted.

## Conclusion

PP with PEUA is associated with increased intraoperative bleeding during CD. Our changes in the management practice for these patients, based on a preoperative assessment, significantly decreased blood loss during CD. Future studies should identify a prompt and an easy diagnostic method for PP with PEUA.

## Supplementary Information

**Additional file 1: Figure S1.** Determination of the horizontal cervix sign.

**Additional file 2: Figure S2.** Typical intraoperative findings in patients with posterior extrauterine adhesions.

**Additional file 3: Figure S3.** Image of a vinyl sheet placed under the patient during cesarean delivery.

**Additional file 4: Table S1.** Patterns of magnetic resonance images according to the presence or absence of findings suspecting PAS.

**Additional file 5: Table S2.** Patient characteristics of placenta previa with posterior extrauterine adhesion before and after practice change.

**Additional file 6: Table S3.** The estimated reasons for posterior extrauterine adhesion.

## Data Availability

The dataset used and/or analyzed during the current study are available from the corresponding author on reasonable request.

## Abbreviations

CD: Cesarean delivery
CI: Confidence interval
IUBT: Intrauterine balloon tamponade
IVF-ET: In vitro fertilization and embryo transfer
IVR: Interventional radiology
MRI: Magnetic resonance imaging
OR: Odds ratio
PAS: Placenta accreta spectrum
PP: Placenta previa
PPH: Postpartum hemorrhage
PTB: Preterm birth
UCS: Uterine compression suture

## References

[1] Rosenberg T, Pariente G, Sergienko R, Wiznitzer A, Sheiner E (2011). Critical analysis of risk factors and outcome of placenta previa. Arch Gynecol Obstet.

[2] Collins SL, Alemdar B, van Beekhuizen HJ, Bertholdt C, Braun T, Calda P, Delorme P, Duvekot JJ, Gronbeck L, Kayem G (2019). Evidence-based guidelines for the management of abnormally invasive placenta: recommendations from the International Society for Abnormally Invasive Placenta. Am J Obstet Gynecol.

[3] Allen L, Jauniaux E, Hobson S, Papillon-Smith J, Belfort MA, Diagnosis FPA (2018). Management Expert Consensus P: FIGO consensus guidelines on placenta accreta spectrum disorders: nonconservative surgical management. Int J Gynaecol Obstet.

[4] Iacovelli A, Liberati M, Khalil A, Timor-Trisch I, Leombroni M, Buca D, Milani M, Flacco ME, Manzoli L, Fanfani F (2020). Risk factors for abnormally invasive placenta: a systematic review and meta-analysis. J Matern Fetal Neonatal Med.

[5] Committee on Obstetric P (2012). Committee opinion no. 529: placenta accreta. Obstet Gynecol.

[6] Eller AG, Bennett MA, Sharshiner M, Masheter C, Soisson AP, Dodson M, Silver RM (2011). Maternal morbidity in cases of placenta accreta managed by a multidisciplinary care team compared with standard obstetric care. Obstet Gynecol.

[7] Licon E, Matsuzaki S, Opara KN, Ng AJY, Bender NM, Grubbs BH, Lee RH, Ouzounian JG, Pham HQ, Brunette LL (2020). Implementation of multidisciplinary practice change to improve outcomes for women with placenta accreta spectrum. Eur J Obstet Gynecol Reprod Biol.

[8] Matsuzaki S, Okada A, Endo M, Nagase Y, Nakagawa S, Hiramatsu K, Kakigano A, Mimura K, Takiuchi T, Tomimatsu T (2019). Horizontal cervix as a novel sign for predicting adhesions on the posterior extrauterine wall in cases of placenta previa. J Clin Med.

[9] Uccella S, Marconi N, Casarin J, Ceccaroni M, Boni L, Sturla D, Serati M, Carollo S, Podesta' Alluvion C, Ghezzi F (2016). Impact of endometriosis on surgical outcomes and complications of total laparoscopic hysterectomy. Arch Gynecol Obstet.

[10] Hwang JH, Lim MC, Joung JY, Seo SS, Kang S, Seo HK, Chung J, Park SY (2012). Urologic complications of laparoscopic radical hysterectomy and lymphadenectomy. Int Urogynecol J.

[11] Suzuki Y, Matsuzaki S, Mimura K, Kumasawa K, Tomimatsu T, Endo M, Kimura T (2017). Investigation of perioperative complications associated with use of uterine compression sutures. Int J Gynaecol Obstet.

[12] Jauniaux E, Collins S, Burton GJ (2018). Placenta accreta spectrum: pathophysiology and evidence-based anatomy for prenatal ultrasound imaging. Am J Obstet Gynecol.

[13] Yang JI, Lim YK, Kim HS, Chang KH, Lee JP, Ryu HS (2006). Sonographic findings of placental lacunae and the prediction of adherent placenta in women with placenta previa totalis and prior Cesarean section. Ultrasound Obstet Gynecol.

[14] Nagase Y, Matsuzaki S, Mizuta-Odani C, Onishi H, Tanaka H, Nakagawa S, Mimura K, Tomimatsu T, Endo M, Kimura T (2020). In-vitro fertilisation-embryo-transfer complicates the antenatal diagnosis of placenta accreta spectrum using MRI: a retrospective analysis. Clin Radiol.

[15] Rac MW, Dashe JS, Wells CE, Moschos E, McIntire DD, Twickler DM (2015). Ultrasound predictors of placental invasion: the Placenta Accreta Index. Am J Obstet Gynecol.

[16] Matsubara S (2014). An easy insertion procedure of Bakri balloon during cesarean section for placenta previa: use of Nelaton rubber catheter. Arch Gynecol Obstet.

[17] Matsuzaki S, Kakigano A, Mimura K, Kimura T (2019). Letter to "Cervical varices unrelated to placenta previa as an unusual cause of antepartum hemorrhage: a case report and literature review": Successful management of postpartum hemorrhage due to cervical varix: modified Matsubara Nelaton method using Bakri balloon. Taiwan J Obstet Gynecol.

[18] Matsubara S, Takahashi H, Baba Y, Usui R (2015). Inserting the Bakri balloon during cesarean section in patients with a narrow cervix: Nelaton method (Matsubara). Acta Obstet Gynecol Scand.

[19] Matsuzaki S, Endo M, Tomimatsu T, Nakagawa S, Matsuzaki S, Miyake T, Takiuchi T, Kakigano A, Mimura K, Ueda Y (2019). New dedicated blunt straight needles and sutures for uterine compression sutures: a retrospective study and literature review. BMC Surg.

[20] Nagase Y, Matsuzaki S, Kawanishi Y, Nakagawa S, Kakigano A, Takiuchi T, Mimura K, Tomimatsu T, Endo M, Kimura T (2020). Efficacy of prophylactic antibiotics in Bakri intrauterine balloon placement: a single-center retrospective analysis and literature review. AJP Rep.

[21] Wing C, Simon K, Bello-Gomez RA (2018). Designing difference in difference studies: best practices for public health policy research. Annu Rev Public Health.

[22] Montalti R, Scuderi V, Patriti A, Vivarelli M, Troisi RI (2016). Robotic versus laparoscopic resections of posterosuperior segments of the liver: a propensity score-matched comparison. Surg Endosc.

[23] Lee H, Kwak C, Kim HH, Byun SS, Lee SE, Hong SK (2015). Diabetes mellitus as an independent predictor of survival of patients surgically treated for renal cell carcinoma: a propensity score matching study. J Urol.

[24] Matsuzaki S, Yoshino K, Endo M, Tomimatsu T, Takiuchi T, Mimura K, Kumasawa K, Ueda Y, Kimura T (2017). Successful anticoagulant therapy for disseminated intravascular coagulation during conservative management of placenta percreta: a case report and literature review. BMC Pregnancy Childbirth.

[25] Sawada M, Matsuzaki S, Mimura K, Kumasawa K, Endo M, Kimura T (2016). Successful conservative management of placenta percreta: investigation by serial magnetic resonance imaging of the clinical course and a literature review. J Obstet Gynaecol Res.

[26] Takahashi H, Baba Y, Usui R, Ohkuchi A, Matsubara S (2018). Video image: Matsubara’s Nelaton and fishing methods for easier Bakri balloon insertion and avoiding its prolapse during cesarean section. Hypertens Res Pregnancy.

[27] Lalani S, Choudhry AJ, Firth B, Bacal V, Walker M, Wen SW, Singh S, Amath A, Hodge M, Chen I (2018). Endometriosis and adverse maternal, fetal and neonatal outcomes, a systematic review and meta-analysis. Hum Reprod.

[28] Uccella S, Manzoni P, Cromi A, Marconi N, Gisone B, Miraglia A, Biasoli S, Zorzato PC, Ferrari S, Lanzo G (2019). Pregnancy after endometriosis: maternal and neonatal outcomes according to the location of the disease. Am J Perinatol.

[29] Miura M, Ushida T, Imai K, Wang J, Moriyama Y, Nakano-Kobayashi T, Osuka S, Kikkawa F, Kotani T (2019). Adverse effects of endometriosis on pregnancy: a case-control study. BMC Pregnancy Childbirth.

[30] Senapati S, Sammel MD, Morse C, Barnhart KT (2016). Impact of endometriosis on in vitro fertilization outcomes: an evaluation of the Society for Assisted Reproductive Technologies Database. Fertil Steril.

[31] Eisenberg VH, Weil C, Chodick G, Shalev V (2018). Epidemiology of endometriosis: a large population-based database study from a healthcare provider with 2 million members. BJOG.

[32] Vercellini P, Parazzini F, Pietropaolo G, Cipriani S, Frattaruolo MP, Fedele L (2012). Pregnancy outcome in women with peritoneal, ovarian and rectovaginal endometriosis: a retrospective cohort study. BJOG.

[33] Kido A, Togashi K, Nishino M, Miyake K, Koyama T, Fujimoto R, Iwasaku K, Fujii S, Hayakawa K (2007). Cine MR imaging of uterine peristalsis in patients with endometriosis. Eur Radiol.

[34] Leone Roberti Maggiore U, Ferrero S, Mangili G, Bergamini A, Inversetti A, Giorgione V, Vigano P, Candiani M (2016). A systematic review on endometriosis during pregnancy: diagnosis, misdiagnosis, complications and outcomes. Hum Reprod Update.

[35] Cali G, Forlani F, Foti F, Minneci G, Manzoli L, Flacco ME, Buca D, Liberati M, Scambia G, D'Antonio F (2018). Diagnostic accuracy of first-trimester ultrasound in detecting abnormally invasive placenta in high-risk women with placenta previa. Ultrasound Obstet Gynecol.

[36] Cali G, Forlani F, Minneci G, Foti F, Di Liberto S, Familiari A, Scambia G, D'Antonio F (2018). First-trimester prediction of surgical outcome in abnormally invasive placenta using the cross-over sign. Ultrasound Obstet Gynecol.

[37] Cali G, Timor-Tritsch IE, Forlani F, Palacios-Jaraquemada J, Monteagudo A, Kaelin Agten A, Flacco ME, Khalil A, Buca D, Manzoli L (2020). Value of first-trimester ultrasound in prediction of third-trimester sonographic stage of placenta accreta spectrum disorder and surgical outcome. Ultrasound Obstet Gynecol.

