# Supplementary figures and images for: Placenta previa with posterior extrauterine adhesion: clinical features and management practice
Source: BMC Surg. 2021 Jan 6;21:10. doi: 10.1186/s12893-020-01027-9 (PMC7789541; doi:10.1186/s12893-020-01027-9)

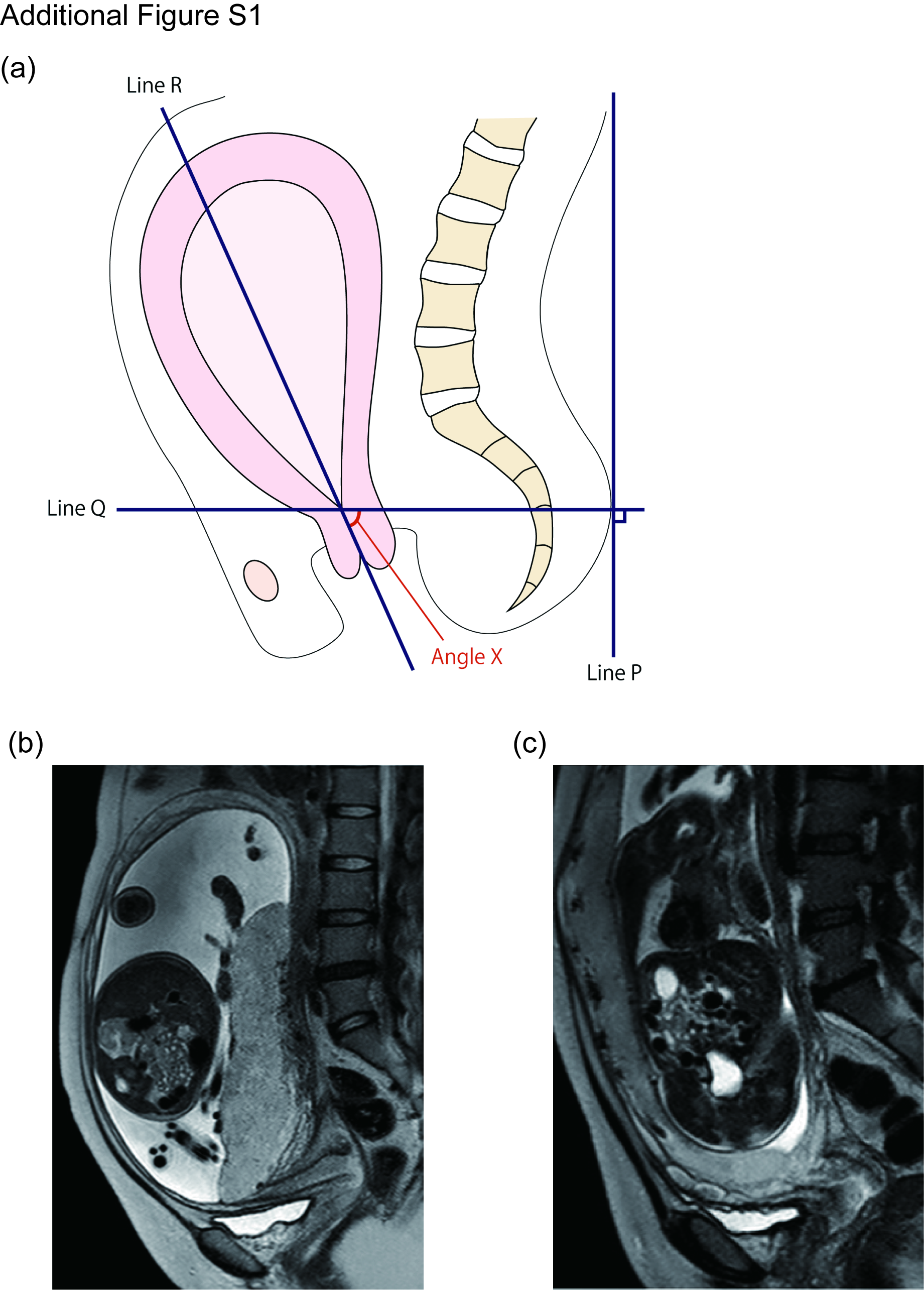

Supplement: Supplementary file 1 — Additional file 1: Figure S1. Determination of the horizontal cervix sign. [file 12893_2020_1027_MOESM1_ESM.tif]

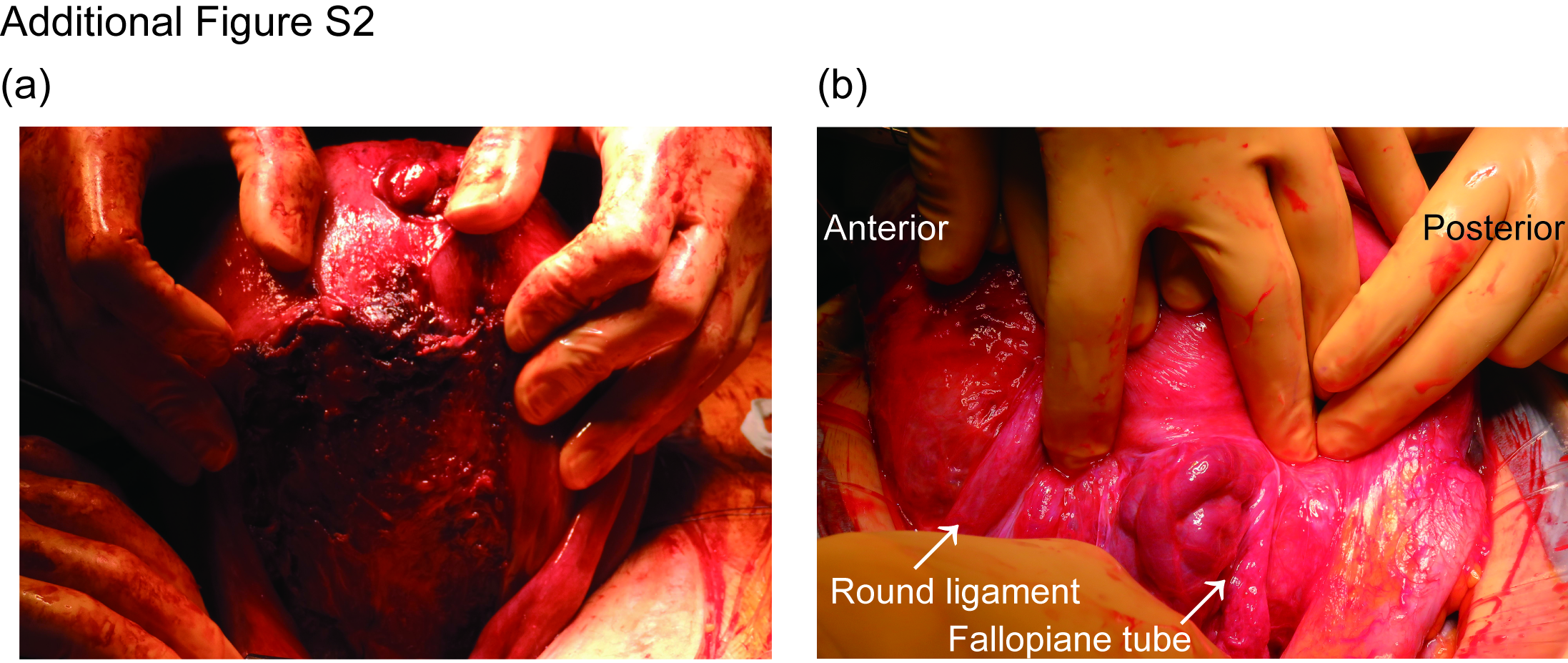

Supplement: Supplementary file 2 — Additional file 2: Figure S2. Typical intraoperative findings in patients with posterior extrauterine adhesions. [file 12893_2020_1027_MOESM2_ESM.tif]

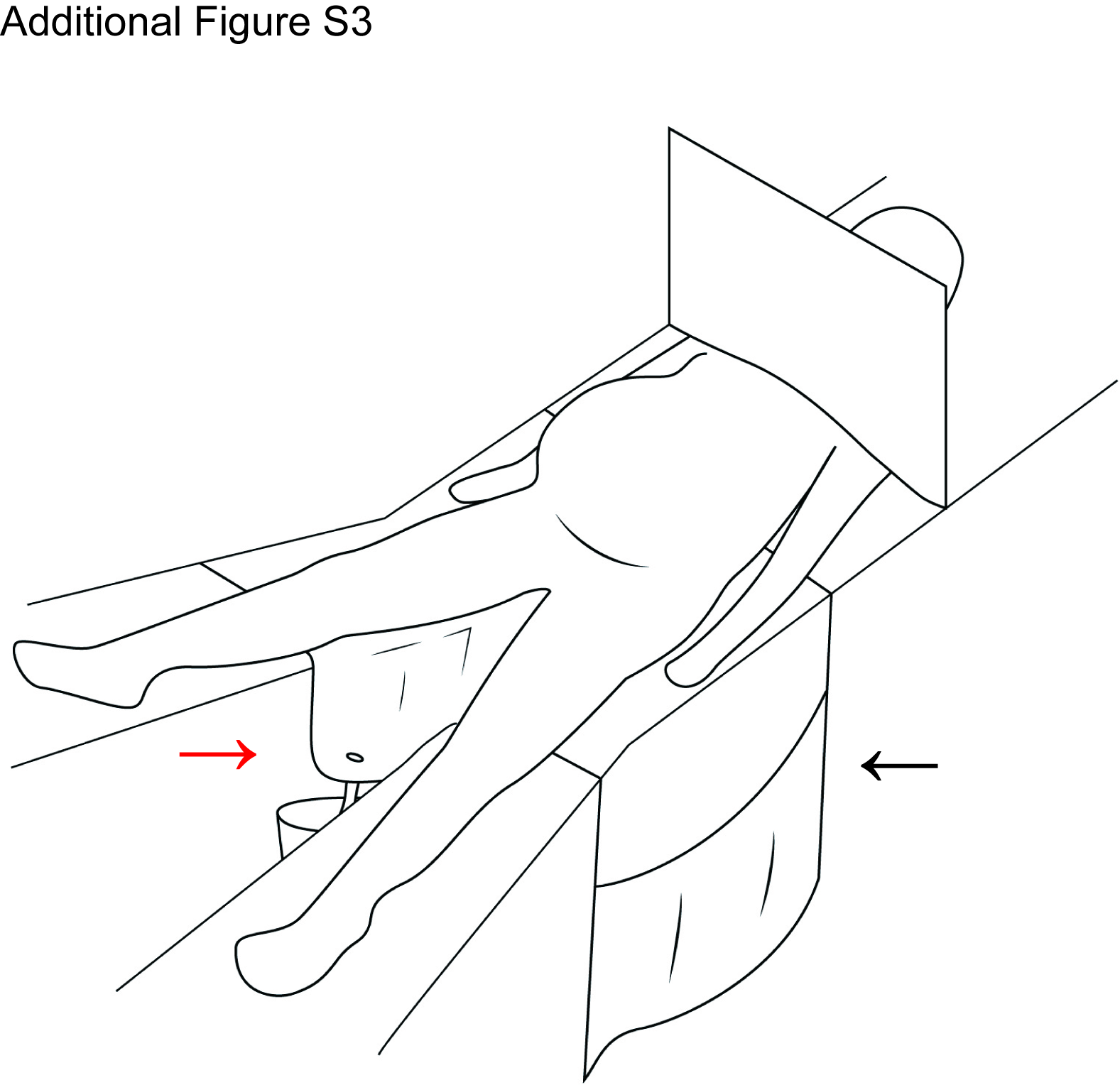

Supplement: Supplementary file 3 — Additional file 3: Figure S3. Image of a vinyl sheet placed under the patient during cesarean delivery. [file 12893_2020_1027_MOESM3_ESM.tif]
