# Supplementary material for: Placenta previa with posterior extrauterine adhesion: clinical features and management practice
Source: BMC Surg. 2021 Jan 6;21:10. doi: 10.1186/s12893-020-01027-9 (PMC7789541; doi:10.1186/s12893-020-01027-9)
Supplement: Supplementary file 4 — Additional file 4: Table S1. Patterns of magnetic resonance images according to the presence or absence of findings suspecting PAS. [file 12893_2020_1027_MOESM4_ESM.docx]

**Additional Table S1. Patterns of magnetic resonance images according to the presence or absence of findings suspecting PAS.**

| Antenatal Diagnosis | Groups | Band | Hetero | Bulging | Others* |
| --- | --- | --- | --- | --- | --- |
| PAS | 1 | Yes | Any | | |
|  | 2 | No | Yes | | Any |
|  | 3 | No | Yes | No | ≥ 1 |
|  | 4 | No | No | Yes | ≥ 1 |
|  | 5 | No | | | ≥ 3 |
| Non-PAS | 6 | No | Yes | No | No |
|  | 7 | No | No | Yes | No |
|  | 8 | No | | | ≤ 2 |

Abbreviations: PAS, placenta accreta spectrum; Band, T2 dark intraplacental bands; Hetero, placental heterogeneity; Bulging, abnormal uterine bulging

* Others indicates myometrial thinning, focal disruption of myometrium, loss of retroplacental T2 dark zone, and disorganized abnormal placental vascularity.

Reused the data from Clin Radiol. 2020 Aug 21:S0009-9260(20)30282-8. Nagase Y *et al.* In-vitro fertilisation–embryo-transfer complicates the antenatal diagnosis of placenta accreta spectrum using MRI: a retrospective analysis/Additional Table S1. Patterns of MR images according to the presence or absence of findings suspecting PAS Copyright (2020) with permission from Elsevier.
