# Supplementary material for: Placenta previa with posterior extrauterine adhesion: clinical features and management practice
Source: BMC Surg. 2021 Jan 6;21:10. doi: 10.1186/s12893-020-01027-9 (PMC7789541; doi:10.1186/s12893-020-01027-9)
Supplement: Supplementary file 5 — Additional file 5: Table S2. Patient characteristics of placenta previa with posterior extrauterine adhesion before and after practice change. [file 12893_2020_1027_MOESM5_ESM.docx]

**Additional Table S2. Patient characteristics of placenta previa with posterior extrauterine adhesion before and after practice change.**

|  |  | Pre | Post | *P* value |
| --- | --- | --- | --- | --- |
| Number of cases | | 12 | 12 |  |
|  | PAS | 1 (8.3) | 0 (0) | 0.99 |
| Maternal age, years (range) | | 34 (27-48) | 34.5 (27-48) | 0.74 |
| BMI at delivery (range) | | 21.8 (19.1-27.2) | 21.7 (19.5-25.6) | 0.49 |
| Parity, Primipara | | 11 (91.7) | 9 (75.0) | 0.59 |
| IVF-ET status | | 4 (33.3) | 5 (41.7) | 0.99 |
| GA at delivery, wks (range) | | 37 (33-37) | 36 (29-37) | 0.62 |
|  | Preterm birth | 5 (41.7) | 7 (58.3) | 0.68 |
| Prior CDs, None | | 11 (91.7) | 10 (83.3) | 0.99 |
| Type of PP | |  |  |  |
|  | Total PP | 8 (66.7) | 8 (66.7) | 0.99 |
|  | Others^a^ | 4 (33.3) | 4 (33.3) |  |
| Location of placenta | |  |  | 0.99 |
|  | Anterior or Central | 1 (8.3) | 0 (0) |  |
|  | Posterior | 11 (91.7) | 12 (100) |  |
| Cervical length^b^, mm (range) | | 56.1 (38.9-68.0) | 56.3 (38.8-66.2) | 0.56 |
| Perinatal complications | |  |  |  |
|  | Preeclampsia | 1 (8.3) | 0 (0) | 0.99 |
|  | GDM | 2 (16.7) | 1 (8.3) | 0.99 |
|  | FGR | 0 (0) | 0 (0) | NA |

Number (% per group) or median is shown.

^a^Others included partial PP, marginal PP, and low-lying placenta.

^b^Cervical length was measured at 27-32 weeks of gestation by transvaginal ultrasound scan.

Abbreviations: Pre, pre-change posterior extrauterine adhesion group; Post, post-change posterior extrauterine adhesion group; PP, placenta previa; GA, gestational age; PAS, placenta accreta spectrum; BMI, body mass index; IVF-ET, in vitro fertilization-embryo transfer; CD, cesarean delivery; GDM, gestational diabetes mellitus; FGR, fetal growth restriction; and NA, not applicable.
