# Supplementary material for: Placenta previa with posterior extrauterine adhesion: clinical features and management practice
Source: BMC Surg. 2021 Jan 6;21:10. doi: 10.1186/s12893-020-01027-9 (PMC7789541; doi:10.1186/s12893-020-01027-9)
Supplement: Supplementary file 6 — Additional file 6: Table S3. The estimated reasons for posterior extrauterine adhesion. [file 12893_2020_1027_MOESM6_ESM.docx]

**Additional Table S3. The estimated reasons for posterior extrauterine adhesion.**

|  |  | Pre | Post |
| --- | --- | --- | --- |
| Uterine exteriorization: yes | | | |
| Suspected endometriosis (Intraop finding) | | 12 | 5 |
| Prior surgery for endometriosis | | 5/12 (41.7%) | 0/5 (0%) |
| For endometrioma | | 4 | 0 |
| For severe adhesion | | 1 | 0 |
| Uterine exteriorization: no | | | |
| Prior surgery for endometriosis | | 0 | 2 |
| For endometrioma | | 0 | 2 |
| Unknown | | 0 | 5 |

Number of cases is shown. Abbreviations: PEUA, posterior extrauterine adhesion; Pre, pre-change posterior extrauterine adhesion group; Post, post-change posterior extrauterine adhesion group; and intraop, intraoperative.
